# Gut Microbiota in Alzheimer's Disease, Depression, and Type 2 Diabetes Mellitus: The Role of Oxidative Stress

**DOI:** 10.1155/2019/4730539

**Published:** 2019-04-17

**Authors:** Maria Luca, Maurizio Di Mauro, Marco Di Mauro, Antonina Luca

**Affiliations:** ^1^Department of Medical, Surgical Sciences and Advanced Technologies “GF Ingrassia”, University of Catania, Italy; ^2^Department of Clinical and Experimental Medicine, University of Catania, Italy; ^3^Department of Biomedical and Biotechnological Sciences, University of Catania, Italy

## Abstract

Gut microbiota consists of over 100 trillion microorganisms including at least 1000 different species of bacteria and is crucially involved in physiological and pathophysiological processes occurring in the host. An imbalanced gastrointestinal ecosystem (dysbiosis) seems to be a contributor to the development and maintenance of several diseases, such as Alzheimer's disease, depression, and type 2 diabetes mellitus. Interestingly, the three disorders are frequently associated as demonstrated by the high comorbidity rates. In this review, we introduce gut microbiota and its role in both normal and pathological processes; then, we discuss the importance of the gut-brain axis as well as the role of oxidative stress and inflammation as mediators of the pathological processes in which dysbiosis is involved. Specific sections pertain the role of the altered gut microbiota in the pathogenesis of Alzheimer's disease, depression, and type 2 diabetes mellitus. The therapeutic implications of microbiota manipulation are briefly discussed. Finally, a conclusion comments on the possible role of dysbiosis as a common pathogenetic contributor (via oxidative stress and inflammation) shared by the three disorders.

## 1. Introduction

### 1.1. Gut Microbiota

Recent technological advances have increased the interest on the relationship between the microorganisms inhabiting the gut (gut microbiota) and human health. The gastrointestinal tract hosts over 100 trillion microorganisms including at least 1000 different species of bacteria [1]. In humans, about 1/3 of gut microbiota is “common,” while the other 2/3 is different from one individual to another, providing our “personal identity” [2]. Despite the difficulties in defining a “good” microbiota, data suggest that, in adulthood, a healthy microbiota is characterized by the community stability and the species diversity. More specifically, despite lifestyle and food changes, the *Firmicutes* (such as Lactobacillus) and the *Bacteroides* represent the main bacterial phyla in the gut [3] followed by *Proteobacteria*, *Actinobacteria* (such as Bifidobacterium), and *Cyanobacteria* [2], which constitute an ecological community entertaining a beneficial relationship with the host [4]. As a result, an imbalance of the intestinal bacteria representation (dysbiosis) could lead to different diseases, ranging from inflammatory bowel disease to obesity, diabetes, and asthma, as well as Parkinson's disease, Alzheimer's disease (AD), and depression [5].

### 1.2. The Gut-Brain Axis

The term “gut-brain axis” refers to a crosstalk between the brain and the gut involving multiple overlapping pathways, including the autonomic, neuroendocrine, and immune systems as well as bacterial metabolites and neuromodulatory molecules [6].

In particular, millions of nerves end in the gastrointestinal tract mucosa, constituting the enteric nervous system which regulates the intestinal functions and communicates with the brain through the vagus nerve. The latter is responsible for the transmission of signals from the brain to the gastrointestinal tract (through the autonomic nervous system) and vice versa [7]. The presence of dysbiosis, causing the breakdown of the intestinal permeability, can lead to an inflammatory condition not limited to the gut, since the proinflammatory cytokines can get into the bloodstream and reach the brain [8]. The importance of inflammation should not be underestimated, since several evidences support its crucial role in several chronic disorders, such as type 2 diabetes [9], AD [10], and depression [11]. Apart from the cytokines, other mediators can send signals from the gut to the brain through the vagus nerve. In fact, especially after a meal rich of fats and carbohydrates, a subgroup of specialized intestinal cells, named enteroendocrine cells, releases hormones and peptides, such as 5-hydroxitryptamine (5-HT), cholecystokinin (CKK), glucagon-like peptide-1 (GLP-1), and peptide YY (PYY) [12]. These mediators exert many important functions. For example, PYY and GLP-1 inhibit the intestinal peristalsis and improve glucose metabolism attenuating pancreatic islet hypertrophy and the insulin resistance [13]. Moreover, these peptides, binding their cognate receptors located in the nucleus of the solitary tract and in the hypothalamus, induce the sense of satiety and modulate the energy expenditure [14]. In addition, GLP-1 seems to be able to upgrade hippocampal neural plasticity improving cognition [15] and to stimulate receptors located in the amygdala and in the hippocampus, thus exerting anxiolytic and antidepressant effects [16].

Interestingly, mice assuming a high-fat diet are more likely to present lower levels of short-chain fatty acids (SCFAs) than rodents assuming a low-fat diet [17]. Moreover, low levels of butyrate have been related with a higher risk to develop type 2 diabetes in humans [18]. SCFAs are involved in neurotransmission, since they modulate the synthesis of several neurotransmitters regulating behaviour and cognition. In particular, both butyric and propionic acid enhance the expression of tyrosine and tryptophan hydroxylase, enzymes involved in the synthesis of dopamine, noradrenaline, and serotonin [19]. Interestingly, evidence suggests that several bacteria could directly produce neurotransmitters, including gamma-aminobutyric acid (GABA) and serotonin. Animal studies demonstrate that Lactobacillus rhamnosus is able to modulate GABA receptor expression in the brain, thus exerting a beneficial role in the treatment of mood disorders [20]. Concerning serotonin, it is mainly produced by the gut enterochromaffin cells and modulates several physiological processes (i.e., mood regulation, sleep, and sexual behaviour). Literature data suggest that Escherichia and Enterococcus are able to produce serotonin both directly and through the production of SCFAs [21].

In summary, gut microbiota and brain are strictly intertwined and communicate through different ways, including the production of bacteria metabolites, cytokines, and neurotransmitters [4, 19, 20]. On this ground, it is not surprising that it has been hypothesized that the gut microbiota could play a pivotal role in the pathogenesis of chronic disorders such as depression, AD, and diabetes.

### 1.3. Microbiota, Inflammation, and Oxidative Stress

Among the various functions of gut microbiota, the regulation of oxidative stress (OS) is probably the most fascinating one [22]. The gastrointestinal tract is rich of sources of nitric oxide (NO), such as intestinal tissues (i.e., mast cells, smooth muscle, and neural plexus), leukocytes, and commensal anaerobes. It has been demonstrated that a high nitrate intake increases both nitrite concentration and NO. In fact, gut lactobacilli and bifidobacteria are able to convert nitrate and nitrite in NO while enhancing the release of NO by host epithelial cells [23]. In addition to NO production from nitrate, gut streptomycetes and bacilli produce NO through their NO synthetase (NOS) from L-arginine [23]. NO is the principal neurotransmitter of the nonadrenergic, noncholinergic enteric nervous system and is released by the activation of NMDA receptors by glutamate. While nanomolar concentrations of NO exert a neuroprotective function being involved in signaling and apoptosis, excessive NO production is noxious, being associated with neuroinflammation, cellular damage, axonal degeneration, and neurodegenerative disorders [10]. An aberrant production of NO leads to detrimental effects due to the generation of reactive oxygen species (ROS), such as superoxide anions (which form the highly reactive peroxynitrite ion, responsible for protein nitrotyrosilation and inhibition of mitochondrial functions) and hydrogen peroxide, which forms the highly reactive hydroxyl radical that is responsible for lipid peroxidation and DNA damage [24]. The main site of the production of ROS and reactive oxygen nitrogen species (RONS) is represented by the mitochondria, primary energy centre involved in the oxidative reactions leading to adenosine triphosphate (ATP) generation. Recent studies have underlined the existence of an intertalk between host and microbiota mediated by mitochondria [25, 26]. Gut microbiota metabolites, SCFAs in particular, influence mitochondrial function reducing ROS production [27]. Other studies have reported that SCFAs are also associated with a reduction of telomere shortening and DNA damage by altering chromatin structures and inducing the production of the antioxidant glutathione [28] and increasing COX-2 activity [29].

If on the one hand, commensal bacteria exert beneficial effects against OS, on the other hand, pathogens (i.e., Salmonella and E. coli) are able to degrade sulphur amino acids leading to hydrogen sulfide (H_2_S) production in the gut. High levels of H_2_S are responsible of several negative effects in the host, such as the inhibition of COX activity and the shifting of the metabolism towards glycolysis, thus leading to an increased lactate and decreased ATP production [30]. Moreover, it has been demonstrated that the exposure to high levels of H_2_S leads to a decreased mitochondrial oxygen consumption and an overexpression of proinflammatory mediator genes, IL-6 in particular [31].

**Figure 1:**
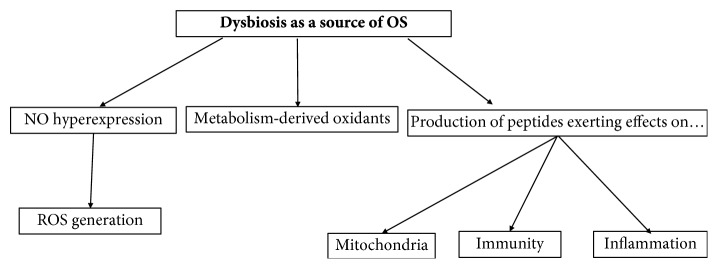
Dysbiosis favors oxidative stress and affects the immunological and inflammatory status of the host. NO: nitric oxide; OS: oxidative stress; ROS: reactive oxygen species.

Considering the important role of the functionally microbiota-derived active substance on host immunological and inflammatory functions, it is apparent that the maintenance of a “healthy microbiota” becomes fundamental for the individual's wellbeing. See Figure 1 for a visual presentation of how dysbiosis favors OS.

## 2. Alzheimer's Disease

Epidemiological studies reported that 50 million people are affected by dementia worldwide [32] with an age-specific incidence ranging from 5 per 1000 at the age 65-70 to 80 per 1000 for individuals older than 85 years [33].

AD is the most frequent neurodegenerative disorders and form of dementia in the elderly [34].

During the early stages of the “typical” form of AD, the main symptom is represented by episodic memory impairment then accompanied by other cognitive domain deficits (visuospatial, attention, language, and executive functions) leading to a loss of abilities of daily living and dementia [35].

To date, thanks to biomarker research, AD is considered a slowly chronic progressive brain disease that can be diagnosed several years before the clinic onset, during a “preclinical stage” [36]. In fact, the pathological hallmarks of AD, including hippocampus atrophy, extracellular amyloid-*β* (A*β*) plaques, and intracellular neurofibrillary tangles of hyperphosphorylated tau protein can be identified up to decades before the occurrence of cognitive decline and behavioural disturbances [37].

Despite some conflicting results, the role of several genetic [38, 39] and modifiable environmental risk factors in the pathogenesis of AD has been documented [40]. Among the modifiable risk factors, special attention has been given to the role of gut microbiome alteration in the maintenance of the chronic age-related low inflammation [41].

### 2.1. Oxidative Stress and Alzheimer's Disease: The Role of Gut Microbiome

From a pathophysiological point of view, as mentioned before, the AD brain is characterized by A*β*1-42 aggregation and neurofibrillary tangles with a consequent immune response activation driven by activated microglia. This immune response activation initially allows A*β* clearance, but, during aging, it undergoes alterations thus leading to a progressive deposition of A*β* plaques which inexorably determines synaptic dysfunction, neuron death, neuroinflammation, and OS [42].

In particular, in the AD brain, several sources of ROS production have been demonstrated: (1) mitochondrial dysfunction determined by a cytochrome c oxidase deficiency [43] as well as by an alteration of their permeability due to the OS-related hyperactivation of glycogen synthase kinase (GSK-3) [44], (2) endoplasmic reticulum dysfunction due to its engagement in the elimination of abundant misfolded proteins, such as hyperphosphorylated tau protein [45], (3) metal ions (i.e., copper, iron, and zinc) accumulated in the neuritic plaques [46], and (4) age-related microglial cell hyperactivation with a subsequent high expression and activation of NADPH oxidase resulting in overproduction of hydrogen peroxide [47].

It should be also emphasized that the CNS is particularly vulnerable to OS for several reasons, including its high oxygen consumption, its use of different reactive species in the signalling process, and its scarce antioxidant metabolism [48].

Although it is still debated whether OS represents the determinant or the immediate consequence of the neurodegenerative processes, OS is indubitably involved in the key events driving the progressive neuronal loss [10]. Markers of lipid peroxidation have been, in fact, detected in biological samples of both AD animal models [49] and patients [50] as well as high levels of protein oxidation markers, such as carbonyls [51]. In this scenario, while a “well-balanced” gut microbiota seems to exert a positive role in the reduction of ROS production via SCFA such as N-butyrate [27], a dysbiosis may lead to systemic inflammation determining, over the years, microglia activation, BBB damage, and consequent crossing of pathogens and immune cells [52].

The most recent theories, in fact, consider AD not only as a result of a confined brain inflammation but also as the consequence of peripheral inflammatory reaction [53]. To support these theories, an association between antimicrobial response and A*β* production has been reported. Indeed, A*β* exerts an antimicrobial peptide role against several pathogens such as bacteria [54] and viruses [55]. It could therefore be hypothesized a periodic, growing with aging, A*β* production following new infections or reactivation of latent brain infections. Moreover, it has been demonstrated that some microbes are able to contribute to A*β* accumulation producing, themselves, a microbial amyloid. The latter, being similar to the cerebral one, easily reaches the brain due to the age-related increased permeability of gastrointestinal mucosa and BBB; being recognized by the immune cells, microbial amyloid determines a massive release of inflammatory cytokines thus sustaining chronic inflammation in AD [41].

Unfortunately, it has been demonstrated that the *age-related reduction of microbiota biodiversity*, with the relative abundance of Proteobacteria and decrease of Bifidobacteria, contributes to the occurrence of dementia not only through the significant reduction of beneficial SCFAs but also through interfering with lipid metabolism. In particular, Bifidobacteria could exert a fundamental hypocholesterolemic role both *directly*, reducing the absorption and production of cholesterol and facilitating its faecal elimination [56], and *indirectly*, increasing the serum levels of leptin, an antiobesity hormone recently associated with hippocampal long-term potentiation and memory impairment prevention [57, 58]. Considering that lipids exert a fundamental role in APP trafficking and processing thus influencing the A*β* oligomer production, the maintenance of microbiota biodiversity appears to be fundamental. Moreover, it should be noted that a dysfunctional lipid metabolism has been found to be associated with anxiety [59], which has been in turn linked to subcortical amyloidosis in nondemented patients [60].

A recent double-blind controlled trial demonstrated that treating AD patients with a probiotic formulation containing Lactobacilli and Bifidobacteria normalized the serum triglyceride levels and improved cognitive performances, thus confirming the role of microbiota in the maintenance of a balanced lipid metabolism [61].

Another mechanism through which microbiota protects against cognitive impairment is represented by the documented ability of Lactobacilli to reduce ammonia concentration in rats [62].

Ammonia is one of the end-products of protein catabolism, historically implicated in the pathogenesis of AD [63]. Due to its neurotoxic nature even at low concentrations, an efficient astrocyte-modulated brain ammonia detoxification through the formation of glutamine by glutamine synthetase (GS) is crucial [64]. With aging, a lower brain GS activity has been demonstrated with the consequent elevation of ammonia concentration. In turn, hyperammonemia causes a progressive mitochondrial dysfunction, thus determining an increase in ROS production and superoxidase levels, while favoring the decrease of cytochrome c oxidase, superoxide dismutase, and glutathione peroxidase [65].

In conclusion, in an era marked by therapeutic failures despite scientific efforts, deepening the knowledge of the diversified mechanisms through which microbiota could exert a role in counteracting cognitive impairment appears to be of fundamental importance. Further research on the effectiveness of microbiota manipulation as a therapeutic tool is needed.

## 3. Depression

Depression is probably the most common mental disorder, affecting more than 17% of the American general population [66]. It is characterized by depressed mood, apathy, anhedonia, sleep disturbances, appetite or weight changes, psychomotor retardation [67] or agitation, cognitive impairment, thoughts of guilt, and recurrent thoughts about death or suicide [68]. This mental disorder, strongly associated with fatigue, loss of productivity, and increased mortality, represents an economic burden for public health [69].

Even if the aetiology of depression is still unclear, several neurobiological mechanisms seem to play a role in its occurrence such as (1) the reduction of serotonin, norepinephrine, and dopamine; (2) the alteration of the hypothalamic-pituitary-adrenal (HPA) axis with the consequently elevated plasmatic cortisol level; and (3) the imbalance between proinflammatory and anti-inflammatory mediators [70]. As discussed below, both animal and human studies have demonstrated a certain role of an “altered” microbiota in this biological scenario.

### 3.1. Gut Microbiota in Depression: The Role of Oxidative Stress

Several studies have reported an imbalance between elevated levels of proinflammatory cytokines, such as IL-1, IL-6, IL-8, IL-12, tumor necrosis factor-alpha (TNF-*α*), and decreased level of anti-inflammatory cytokines, such as transforming growth factor-beta and IL-10 in patients suffering from depression [70, 71]. Moreover, nonspecific inflammatory markers (i.e., acute phase protein, *α*1-antitrypsine, haptoglobin, fibrinogen, and C-reactive protein) have been found to be high in depressed patients [72].

From a biochemical point of view, as mentioned before, the activation of the inflammatory pathway is characterized by a hyperproduction of reactive oxygen species (ROS) and reactive nitrogen species (RNS) with a consequent damage of DNA, proteins, mitochondria, and cell membranes [73]. The presence of an oxidative and nitrosative stress is supported by the detection in depressed patients of high levels of by-products of lipid peroxidation such as malondialdehyde and 4-hydroxynonenal [74]. Moreover, if on the one hand, depression is characterized by an increased oxidative and nitrosative stress pathway, on the other hand, endogenous antioxidants such as zinc, glutathione, coenzyme Q10, melatonin, and vitamin E have been found to be decreased. These aforementioned substances, involved in the mitochondrial functioning and in the regulation of cAMP/circadian gene, may lead, if deficient, to neurodegeneration and decreased neurogenesis and neuroplasticity [75]. Probably, the major expression of an “altered” gut microbiota lays just in these oxidative mechanisms. In fact, while germ-free mice present a reduced antioxidant enzyme activity (i.e., catalase, glutathione peroxidase, and superoxide) [76, 77], an altered microbiota can stimulate the NADPH oxidase [78] and the NO synthesis [79], thus inducing OS.

The “depression-associated bacteria” are able to induce depressed mood both directly, producing valeric acid (adenosine A1 receptor inverse agonist) [80], and indirectly, promoting the production of kynurenine from tryptophan [81].

Moreover, while in physiological conditions, gut microbiota is separated from the systemic immune system by the epithelial barrier, in pathological conditions, it can reach the mesenteric lymph nodes determining the activation of monocytes and macrophages and the consequent production of inflammatory mediators with antibacterial properties, such as lysozyme [82].

In normal conditions, the gut epithelial barrier is protected from OS and inflammation by a class of ubiquitously expressed intracellular proteins, the heat shock proteins (HSP). These proteins are chaperones and play a role in the synthesis and folding of other proteins [83].

Due to their role in the repair and stabilization of proteins, evidence suggests that the synthesis of the HSPs is strongly enhanced in physical and psychological stressful conditions [84]. Nevertheless, if on the one hand, the upregulation of the HSPs can be considered as a cellular “defence” mechanism, on the other hand, their release in the extracellular matrix, occurring during cellular necrosis or apoptosis, can be remarkably harmful. Extracellular HSPs, in fact, are able to stimulate an inflammatory response leading to an increased proinflammatory cytokine secretion [84]. The demonstration of high plasmatic concentration of extracellular HSP70 in depressed patients has led to hypothesize that it could play a role in the occurrence of mood disorders [85]. Interestingly, the physiological epithelial HSP tone is influenced by gut microbiota activity and diversity. Several Bifidobacteria and Lactobacilli are, in fact, strong inducers of gut epithelial HSPs, thus contributing to gut protection [86].

The relationship between depression and microbiota has been known for several decades, when animal studies reported that stressed mice presented marked reductions of the number of gut lactobacilli [87]. Recently, human studies have confirmed this early observation, reporting a less represented microbial diversity, with a relative abundance of *Bacteroidetes* and a reduction of *Lachnospiraceae*, in depressed patients [88]. In addition, chronic depressed subjects present high plasmatic levels of immunoglobulin (Ig) A and IgM antibodies against the LPS of *Enterobacteriaceae*, thus supporting the hypothesis that microbiota could play a role in the occurrence of depression probably sustaining a chronic inflammatory status [82]. Moreover, stressed germ-free mice present high circulating levels of ACHT and corticosterone, depression-sustaining hormone [89].

On these grounds, even if literature data still report conflicting results, the interest in treating depression through the administration of probiotics is growing so that the term “psychobiotics” is increasingly used. However, despite probiotics are able to decrease the plasmatic levels of cortisol with a consequent psychological wellbeing and a reduction of depressive symptoms [90], literature data are still inconsistent. In fact, while some studies demonstrated the efficacy of probiotic augmentation in the reduction of depressive symptoms in drug-resistant patients [91, 92], the administration of probiotics alone seems to be ineffective [93].

Nonetheless, it is undeniable that the potential usefulness of probiotics in major depression is a fascinating and worthy of investigation topic, also considering the elevated tolerability of these compounds.

## 4. Type 2 Diabetes Mellitus

Diabetes and obesity, two world epidemics, represent a global challenge for health care [94].

The 2016 *Global Report on Diabetes* by the World Health Organization (WHO) states that in 2014 the prevalence of diabetes reached up to 8.5% in the adult population, while more than 1 in 10 adults were obese [95]. Diabetes mellitus was the 7^th^ cause of death in 2016 and is projected to move up in the rankings by 2030 [96, 97].

Hence, research pertaining obesity, metabolic syndrome, and type 2 diabetes mellitus (T2DM) has been focusing on the identification of potentially modifiable dysfunctions, among which dysbiosis of the gut microbiota has been attracting much interest [98–101].

As previously stated in this review, gut microbiota plays a fundamental role in physiological and pathophysiological processes occurring in the host and it is apparent how diet could directly influence the fine balance of the intestinal ecosystem [102–105].

### 4.1. Gut Microbiota in Obesity, Insulin Resistance, and T2DM: The Role of Oxidative Stress

Obese individuals are frequently characterized by insulin resistance, a condition associated with low-grade subclinical inflammation leading to hyperglycemia and favoring the onset of T2DM [106].

An animal-model study demonstrated how gut microbiome transplant from twin mice discordant for obesity to germ-free mice influenced the metabolic arrangement of the host. More specifically, mice receiving faecal transplant from obese donors had a higher tendency to develop obesity compared to mouse receiving the transplant from a lean donor. Interestingly, diet modulated the colonizing of the microbiome and acted as a protector from developing the obesity phenotype [107].

Differences between obese and lean people both in terms of concentration and type of resident bacterial population have been reported. Overall, individuals with a low bacterial richness are characterized by marked adiposity, insulin resistance, and dyslipidaemia [108]. *Firmicutes* and *Bacteroides* phyla account for the 90% of the adult gut flora. An imbalance between the two species has been linked to obesity with conflicting results, but it seems that a reduced production of the microbiota-induced fermentation product butyrate (exerting anti-inflammatory and antioxidative properties and increasing insulin sensitivity in mice) and an increased release of LPS (already described as endotoxin) could favor obesity; the latter is characterized by a microbiota prone to harvest energy from diet [109, 110].

Concerning T2DM, gut alterations lead to an enrichment in membrane transport of sugars, branched-chain amino acid transport and sulfate reduction, decreased butyrate biosynthesis, and OS response that could account for the proinflammatory state characterizing diabetes. Interestingly, patients suffering from T2DM show a dysbiotic condition characterized by a decrease of butyrate-producing bacteria (such as Firmicutes, Roseburia intestinalis, and Faecalibacterium prausnitzii) and an increase in opportunistic pathogens. Despite this unfavorable condition, the gut environment of T2DM attempts to limit OS, with an increase in functions related to OS resistance, such as catalase, peroxiredoxin, and glutathione reductase. In general, despite the role of some metabolites (such as butyrate) needs to be further confirmed, it has been hypothesized that dysbiosis in T2DM could be related to qualitative, more than quantitative, changes in the gastrointestinal ecosystem and a condition of OS that could increase the risk of diabetes complications [101, 111].

While scientific evidence supports the link between gut microbiota and metabolic syndrome [100], the relationship between inflammation, gut microbiome, and metabolic alterations has not yet been clearly elucidated.

A potential interconnection between these three factors could be represented by diet; indeed, it has been reported that a high-fat diet leads to an increase in the proportion of lipopolysaccharide- (LPS-) containing microbiota and in the LPS plasma levels, thus leading to the so-called endotoxemia. The latter contributes to the occurrence of insulin resistance and accounts for chronic low-grade inflammation and OS characterizing metabolic syndrome. The circulation of endotoxins is made possible through the permeability alterations and the changes in composition affecting the gut [112, 113]. LPS directly sustain insulin resistance and inflammation via toll-like receptor (TLR) signaling. TLR4 in particular serves as a coreceptor for the monocyte differentiation antigen CD14 and mediates the LPS-induced inflammatory cascade and innate immune response [113, 114].

The discussed findings have practical implications. In fact, it can be inferred that the manipulation of intestinal ecosystem could interfere with the dysbiosis-related prooxidative and proinflammatory mechanisms described above. Preliminary reports support this hypothesis. In fact, the use of antibiotics in mice has been related to reduced endotoxemia, OS, and inflammation [115]. In addition, *Lactobacillus*, *Bifidobacterium*, and *Enterobacter halii* are bacterial candidates for the treatment of obesity, even though more studies are needed to confirm their efficacy [116]. In terms of future perspectives, the characterization of faecal metagenome could help identifying people at high risk of developing metabolic and inflammatory complications [18].

## 5. Gut-Brain Axis: Therapeutic Perspectives

As stated above in the sections dedicated to AD, depression, and diabetes, several studies have reported the usefulness of microbiota manipulation in the treatment of these disorders [61, 91, 115]. In recent years, special attention has been given by researchers to probiotic supplementation with promising results [117–119]. Despite further insights on the efficacy of dysbiosis restoration in these disorders are needed, the gut-brain axis cannot be simply dismissed as a “fashionable topic” [120].

## 6. Conclusion

Dysbiosis has been demonstrated to exert regulatory functions on inflammation and OS and represents a pathogenetic contributor shared by AD, depression, and T2DM [8, 22, 41, 81, 101], three disorders characterized by a prooxidative and proinflammatory condition [42, 70, 111]. The gut-brain axis can account for the molecular similarities linking these disorders, also confirmed by the high rates of comorbidity between depression and T2DM, which in turn increase the risk of dementia. Metabolism, cognition, and mood are strictly intertwined; if glucose toxicity can directly interfere with the cognitive functions, the insulin pathway is involved in amyloid formation, while depression can precipitate neuronal damage through inflammatory mechanisms [121]. Deepening the knowledge on the pathogenetic mechanisms of these burdening disorders could open new scenarios. In fact, the manipulation of the gut environment could be further investigated as a preventive and/or therapeutic tool with (potentially) a good safety profile.
